# Supplementary material for: Navigating life when a loved one’s (euthanasia) death is near: A narrative interview study from the Netherlands
Source: PLoS One. 2025 Aug 1;20(8):e0327917. doi: 10.1371/journal.pone.0327917 (PMC12316235; doi:10.1371/journal.pone.0327917)
Supplement: S1 File — (DOCX) [file pone.0327917.s001.docx]

**Supplement 1**

| *Vignette 2: Navigating hospitals and (care) homes, past and present*  Both Christopher (64, several children and grandchildren, retired garage-owner) and his wife had to navigate different hospitals and a nursing home as his wife’s death approached. Over the course of several years, his wife’s condition had declined because of pain related to her metastasized cancer combined with shortness of breath due to pre-existing heart problems. They had promised to “help each other until the end.”  Christopher and his wife had already seen many deaths among their siblings, including euthanasia deaths. His wife had also already spoken about euthanasia at an early stage. “She had already agreed with the GP that if…if it would become really bad, she wanted euthanasia.” “She said ‘I don’t want to suffer, you shouldn’t see me waste away, I don’t want that’.”  At one point, his wife was admitted to one hospital, while Christopher had to undergo emergency-treatment for a myocardial infarction in another hospital. They were both discharged to the same recovery home, which was a difficult experience for them both. They had to support each other while recovering themselves, and experienced insufficient support from the nursing-home staff. Christopher explained how these experiences had been “decisive” for his wife to pursue euthanasia. “She says, to be so reliant on the help of others, of outsiders (…) you can’t do anything yourself anymore.” Once they returned home again, Christopher and his daughters, together with home care once a day, took care of his wife.  The GP, who they had known for 32 years and with whom his wife had discussed her euthanasia-request, unexpectedly retired. A new, younger GP took over the practice and he “followed their story closely” and was “careful” in his handling of conversations about euthanasia. Christopher remembers how the GP as well as the oncologist and cardiologist had called it “a brave decision (…) and really understandable”. Christopher’s wife had initially wanted to receive the euthanasia on the death anniversary of her sister, but it happened earlier, because she could not endure the pain and tiredness anymore.  “Fierce” and “heavy” is wat Christopher says, about the complete experience of his wife’s illness-trajectory and death; the images of the fire at the crematorium stick with him as well. He noticed that more psychological support for his wife would have been desirable, but also that his wife probably wouldn’t haven’t accept it, given bad experiences with psychologists in the wake of a traumatic event in her younger years. For himself, he says that coping with the loneliness is the most difficult thing now. “There are lots of places I could go for help, but I don’t want to. I don’t want to be a burden.”  *Vignette 3: Navigating hospitals and (care) homes, past and present*  Katherine (76, widowed, two children, retired social worker) describes how peaceful the final moments of her sister’s life had been because of the euthanasia death taking place at Katherine’s home; how special this had been given her sister’s long-lasting anxiety; and how they both had feared an in-hospital death because of previous experiences during hospitalizations of themselves and beloved others. | | |
| --- | --- | --- |
|  | | |
| *Vignettes 4: Unexpected events & professional care unfolding in different ways*  Sophia (54, several children, higher manager in education) and her husband prepared themselves well for his impending death, by talking with each other and with their GP about his wish to die at home. She also ensured help from a befriended community nurse. But her husband’s condition rapidly and unexpectedly worsened. Also, their GP went on vacation just when they needed her most.  *Vignettes 5: Unexpected events & professional care unfolding in different ways*  Peter (73, retired psychologist) had fully supported his wife’s decision for euthanasia, but she died at home several days before it was scheduled. The moment they learned of his wife’s metastasized ovarian cancer and the poor prognosis was very difficult for Peter. “Mentally it was very difficult for me.” “I thought, this just can’t be true. But it was!”. Peter and his wife held shared views on life: they were “for self-autonomy,” “being in control of one’s own life and death.” Therefore, it felt logical that his wife, as soon as she heard about her metastasized ovarian carcinoma, decided she would want euthanasia. Peter describes how he and his wife were able to openly speak with each other about it all. He knew from a previous euthanasia-death in their family that that it could be a good and very carefully monitored process, but nevertheless it felt “confrontational” and “something very strange” to set a date and time to die in advance and to be a witness of the sudden death.  Due to palliative systemic treatment, his wife’s life was extended for longer than predicted, but the disease further progressed, and she decided not to undergo further treatment. It all became too burdensome. His wife was about to receive care at home until her death under supervision of their GP. “We always had contact with the GP, and the relationship between my wife and the GP had always been good over the years.” However, Peter’s wife broke her leg due to an accident, and Peter could not manage the daily care anymore. Their GP swiftly arranged a place in a hospice nearby, where Peter’s wife stayed for another couple of months and received supportive care from a physiotherapist and a social worker, among others.  Peter describes how several times in the hospice, his wife described her excruciating tiredness and said she wanted to schedule the euthanasia. “She thought, if it goes on like this, I can’t take it anymore. But after a few days it usually got better”. After conversations with both the GP and Peter they postponed the euthanasia several times. Eventually his wife went home for what would become the last week of her life. Apart from the GP, they had support from Peter’s sister who stayed with them. At home, his wife had a mild epileptic attack several days before she was about to receive euthanasia; she received some emergency sedatives from the GP, and died in her sleep not long after that. “I’m happy that it happened like this instead of having to wait for an appointed moment [for euthanasia]” says Peter about it and adds that he is happy with the care they received throughout the whole trajectory. | |  |
|  | |  |
|  | |  |
| *Vignette 6: Strong emotions related to “ghosts of the past” & concerns about the future*  Cliff (69, several children, retired architect) learned over time that the overwhelming emotions triggered by his wife’s metastasized lung cancer and life-threatening complications caused by it, had to do with the traumatic death of his mother 50 years prior. “That gets carved into your life, you can’t get rid of it.” He experienced enormous tension, anxiety, and sleeplessness. “I was falling apart from the stress (...) Rationally you can explain it, but it feels extremely overwhelming.”  He found help from a psychologist, although he initiated the referral himself. And while he knows his wife is looking ahead and thinking about the last phase of life, he is not as inclined to do so. “Once you are there, reality is often different.” However, he does worry about access: “I want to avoid the situation where we think, now we really need to do something, but you don’t have access [to professional care].”  Several times, he and his wife had experienced difficulties with “getting into the circuit” when his wife suffered from complications like severe pain. They had difficulty being helped by various locum GPs in their family practice and were once turned away at the out-of-hour GP-service before finally being treated in the hospital ER. Cliff hopes to find support from his wife’s GP -which is a different one than his own- on such occasions in the near future. But, he says, “The difficult thing is that a lot of medical emergencies happen at night.”  *Vignette 7: Ambivalent feelings after the euthanasia-death of a parent*  Ryan (31, geologist) was well prepared for the euthanasia-death of his father and it fit with his family’s life views. Still, it overwhelmed him, due to the speediness of the euthanasia-death and specific timing of events. |  |  |
|  |  |  |
| *Vignettes 8: Caring for the other, caring for oneself, and the possible place of euthanasia therein*  Christina (75y, two children, retired administrative assistant) after several years still has difficulty coming to terms with her husband’s euthanasia-death. She struggles with how it has impacted her so negatively while she was sure that “he never would have wanted to hurt me,” given how caring he had been for her over all those years. She is also puzzled by how differently she and her husband seemed to have experienced these last moments of his life. But, she adds, “you can never know all about another person, even if you have been together for 40 years” and wonders whether her husband’s specific emotional reactions had to do with his previous brain-injury due to an accident.  Christina’s husband had been diagnosed with stomach cancer after a long time of tiredness, declining physical condition and non-conclusive diagnostic tests. He was admitted to the hospital, underwent explorative surgery and a medical specialist concluded that there were no treatment-options left. Once back home, her husband’s condition rapidly declined. She says that he refused any homecare and that she was happy to take the daily care upon her despite the home “changing into a hospital” and becoming “personally threadbare and broken” herself. “You keep caring for each other”. She also knew what it could be like: she had previously cared for her mother dying at her home and had lost her father and brother.  Christina and her husband had agreed that he “didn’t need to bear it out until the bitter end” and her husband chose to pursue euthanasia. However, their own GP left for pregnancy leave and they had to ask another GP whom they did not know to step in. The new GP frequently visited them in the following weeks to get to know them and to get a good impression of the euthanasia-request and her husband’s situation. Eight weeks after the diagnoses of incurable cancer, the assisted death took place at their home.  Christina uses the phrases “something unreal” and “horrible” for her husband’s death. She describes how he was still talking to her at the moment when he received the injections and then suddenly died. “Talking one moment, dead the next!” “That morning I gave him a cup of coffee and a toast; a half hour later he was gone. That’s so strange!” She contrasts it with the death of many others she had accompanied as a volunteer in a nursing home, who had slowly “drifted out of life”.  She has no siblings or close friends to go to with these experiences, and she does not want to burden her children. She had been very happy though with the practical help on financial and administrative matters by the funeral undertaker, which she had not really thought of in advance. She values the continuing care of the GP who carried out the euthanasia and has spoken with him on multiple occasions. Nevertheless, she finds it difficult to express her continuing discomfort. “Then he [the GP] answers, ‘but for him [the husband] this was the best outcome’”.  *Vignettes 9: Caring for the other, caring for oneself, and the possible place of euthanasia therein*  Ashley (32 years, sales assistant retail) had been closely involved in the daily care of her father together with her sisters, which had been intense. And she had learned how he had great concern for them all up until the final moments before his euthanasia-death. |  |  |
|  |  |  |
| *Vignette 10: A lot to navigate, missing the past GP*  Charlotte (49, several children, freelance researcher in the social sciences) has to navigate overwhelming emotions related to her husband’s illness, uncertainty regarding her income, and tensions that arose in the relationship with friends and relatives. She tells about how she misses her past GP who knew her (family’s) story and about the discontinuity of GPs’ care in their area. *See*  *Vignette 11: A lot to navigate, but finding support from each other, friends, one well-known GP and a specialized palliative homecare team*  Marc (60, several children, CEO large company) and his wife first and foremost took care of each other, could count on their own network, and unexpectedly found great support from their GP together with a specialized palliative homecare team. “We didn’t know that dying at home could be such a meaningful experience and that you can receive such good home care”  Marc tells – often using the pronoun ‘we’ – about how he and his wife had navigated different hospitals, experimental treatments, and some serious complications, with help of a befriended oncologist among others. His wife’s lifespan had been extended beyond what was expected from her tumor-type. “If I didn’t have you I’d have been dead long ago” his wife had told him.  However, a moment came when no treatment options were left, and their GP took over “who knew about every step we had taken so far”. Marc tells about how their GP started visiting them weekly at home and initiated the care by a specialized palliative home care team. The coordinating nurse of the team started to visit them frequently as well, daily supportive care for his wife was initiated, and they could contact them with any question or concern.  During treatment, Marc’s wife had not wanted to speak about the end of life, because she had needed to remain positive. But once at home they slowly started talking about it. And they started talking about euthanasia first, not about other care-options at the end of life such as palliative sedation. “We didn’t like that word, palliative”, Marc explains, and adds how it reminded them of his mother in law’s death. Marc’s wife started to discuss euthanasia with their GP, something that Marc found quite emotional. “It makes your heart shrink to hear something like that from your partner. I thought, oh no, not that.” He really wanted to support her, “you’re the director of your own life”. Nevertheless, he found the prospect difficult. “In my mind I heard the doorbell, I could see the doctors coming in.” In the end his wife did not chose to pursue euthanasia. “I think she did that to protect us.”  During the last weeks, both for medication-interventions as well as just keeping an eye on them, they could rely on the almost daily visits of the GP and swift action of the nurses of the palliative care team if necessary. In the end, his wife died peacefully at home with hardly any medication, in the presence of Marc and their children. “That time was so precious to us, it was so intense, because we knew we were saying goodbye to each other.” Both the GP and the coordinator of the palliative care team visited him afterwards several times to talk things through, which Marc greatly appreciated. |  |  |
|  |  |  |
