# Supplementary material for: Navigating life when a loved one’s (euthanasia) death is near: A narrative interview study from the Netherlands
Source: PLoS One. 2025 Aug 1;20(8):e0327917. doi: 10.1371/journal.pone.0327917 (PMC12316235; doi:10.1371/journal.pone.0327917)
Supplement: Attachment — Submitted filename: Response to the reviewers.docx [file pone.0327917.s003.docx]

Response to the editor and reviewers

| **Comments to the editor** | |
| --- | --- |
| 1. Please ensure that your manuscript meets PLOS ONE's style requirements, including those for file naming. | We have checked everything. |
| 2. In the online submission form, you indicated that your data is available only on request from a third party. Please note that your Data Availability Statement is currently missing contact details for the third party, such as an email address or a link to where data requests can be made. Please update your statement with the missing information. | The data are only available on request because of the privacy of the respondents. Data requests can be made by writing to one of the co-authors: Prof C. Leget, [c.leget@uvh.nl](mailto:c.leget@uvh.nl). We will indicate that the data are available only with restrictions |
| 3. Please include captions for your Supporting Information files at the end of your manuscript, and update any in-text citations to match accordingly. Please see our Supporting Information guidelines for more information: <http://journals.plos.org/plosone/s/supporting-information>. | We have checked everything. |
| **Comments to reviewer #1** | |
| 1. Occasionally there are two spaces or lack of a full stop or a space between the last word and the full stop, but I suppose the final editing will solve this | We tried to remove the extra spaces ourselves and trust the final editing will address any we might have missed. |
| 2. Why is there such a big variation in interview duration (27-98min) | There was a wide range in interview duration. Some interviewees were more concise in response to verbal prompts and follow-up questions, less detailed in their storytelling, or less spontaneous in their narration. This, however, does not mean the content of their answers was less rich or to the point. Possible causes may be educational level, ‘verbal story-telling capacity’, or a more pragmatic outlook on life. We did not have the impression that felt unsafe or inhibited when telling their stories. In further research we could use other types of prompts and methods (observational, visual, audio) for story- eliciting and -telling. We have discussed this issue in the limitations. |
| 3. How did you get the interviewees to talk about euthanasia if BR did not actively ask about euthanasia if the participants did not explicitly mention it? | We agree with both reviewers that this procedure requires more attention. We adjusted the paragraph in the methods section and reflect on the issue in the discussion of the revised manuscript. |
| 4. Some sentences that seem to be incorrect:  “BR did not actively ask about euthanasia IT during the interview if participants did not first explicitly mention it themselves because of research-ethical considerations” | This sentence has been deleted. |
| With regard to “undue influence” of relatives on patient’s decisions, we could as how both patients and relatives can be sufficiently supported throughout all dimensions of life when death is near | We have changed this sentence. |
| Why did gender seem to be such an important parameter ? | Because it was not our focus of research, we do not have evidence that this is an important parameter. Our intuition and experience, however, is that gender does play a role in doctor-patient interaction. In order to be true to what we have researched, we have changed the wording of the sentence from ‘important parameter’ to ‘possibly a parameter of importance, in need of further inquiry’. |
| Vignette 6: what is GP surgery? | In this sentence, we were referring to a collective group of general practice healthcare professionals. After consulting with the native English speakers on our team, we determined that ‘family practice’ would be the most appropriate term to use here. |
| I feel like the conclusion might still be a bit stronger, with perhaps a firmer recommendation towards the health care professionals: they can play a big role in the end-of-life care, but they are only one part of the whole process that a patient and their relatives are confronted with. The difficult situation for the relatives does not end after the patient has died. And I really loved how the article described the situation as being 'multilayered', that term could also be used in the conclusion. (just a suggestion). | Thank you for these suggestions. We agree that the multilayered aspect of end-of-life care can be reiterated in the conclusion and have now done so. We have also added a sentence about the fact that end of life negotiations and concerns continue for relatives even after a loved one has died. |
| **Reviewer #2** | |
| 1. The study focuses solely on cancer patients, but the rationale for this selection remains unclear. It should be explained already in the background section together with the characteristics of this patient group, e.g. in comparison to neurological patients. Their significance for the transferability of the results to other disease groups should be discussed later. | In the Netherlands patients with incurable cancer constitute the largest group of those requesting euthanasia (57.8%)—as we mention already in the introduction. These are also perceived to be the least complicated cases, both from a judicial perspective and from a social perspective. For these reasons, we decided to start with this group when renewing our exploration of relatives’ experiences.  We hope that further research can determine if and to what extent our findings are transferrable to other patient groups. Relatives of patients with other illnesses, for instance neurological (ALS, dementia), cardiological, or psychological in nature may have even less predictable trajectories than the ones our respondents described. Furthermore, the mental faculties of these patient could be affected, which further complicates navigating the different dimensions we outline in our results. Families’ navigating strong emotions in psychiatric illness and ‘voltooid leven’ (tired of living) cases may have specific qualities as compared to euthanasia in patients who are terminally ill as well.  We have added this reflection to the limitations section of the manuscript. |
| 2. Page 3: This narrative interview study showed us that…The results of the narrative interview study invited us to shift our perspective: … we may consider the perspective that it is GPs and other professionals who get involved in death and dying as a family affair - These are results of the study, which I would see in the corresponding section, rather than in the introduction. | We have moved these sentences to the beginning of the results section. |
| 3. Page 3: The research question that guided this sub-study was: what can we learn from co-constructed interview narratives about the needs and felt responsibilities of cancer patients’ relatives during euthanasia decision-making in the broader context of end-of-life care in the home-setting? In particular, we were interested in how relatives’ needs and responsibilities take shape in interaction with their GP. - This text should be located at the end of the introduction. | We relocated these two sentences to the end of the introduction. |
| 4. Page 3: The study was conducted as part of a larger research-project (50 interviews in total) exploring the needs and responsibilities of patients, GPs and other professionals working in primary care  - Did this study focus on euthanasia? Please specify. | We clarified this by changing the sentence into:  The study was conducted as part of a larger research-project (50 interviews in total) exploring the needs and responsibilities of patients, GPs and other professionals during end-of-life decision-making and care, including euthanasia, in Dutch general practice. |
| 5. In The Background you write: … we decided to further explore family involvement in the Dutch practice of euthanasia in the broader context of end-of-life care at home under guidance of GPs, by performing a narrative interview study. And then in the Methods section: From the information leaflet and conversation with BR, the participants knew that euthanasia could be one of the topics in the interview. However, in the prospective group, BR did not actively ask about euthanasia it during the interview if participants did not first explicitly mention it themselves because of research-ethical considerations, even though euthanasia is widely spoken about in the public sphere (see subsection Dutch context and definitions).  - This procedure and its impact on the results should be discussed methodically. | We agree with both reviewers that this procedure requires more attention. We adjusted the paragraph in the methods section and reflect on the issue in the discussion of the revised manuscript. |
| 6. Results Page 8: … it was remarkable to see how many interviewees had to deal with acute and chronic health issues of their own while caring for their ill relatives, … - I would replace “many” with ‘some’ or something similar, as many is very quantifying in a qualitative study. Same for “frequently and spontaneously referred to navigating workplaces…” à Better use e.g. „several“. | We have changed both words to ‘several’. |
| 7. It is not transparent if statements come from the prospective or the retrospective interviewee group. Why? This procedure is not justified or discussed. | The results presented are relevant for both the prospective and retrospective groups, unless otherwise stated. We have added a sentence to the beginning of the results section to clarify. |
| 8. It also becomes not clear, how the experiences of euthanasia and regular assisted dying are identical or if there are possibly differences. | The goal of this study was not to compare euthanasia to other forms of death (for example, palliative sedation). We acknowledge that there may be some overlap in relatives’ experiences in various end-of-life scenarios. For instance, it’s likely that relatives have a range of positive and negative experiences like the ones we describe in this study. |
| 9. The results section is very long: in my opinion, the descriptions of the main topics could benefit from cutbacks. What are the key messages? Is it possible to illustrate them with tables or figures? | To reduce the length of the results section, and to address the following comment, we have decided to move all the vignettes except for the first to the supplementary file. We have explained this in the text as well. |
| 10. It is sometimes difficult to understand how the statements relate to the data. I would suggest adding all vignettes to the manuscript as an appendix and only inserting short summaries, indirect or direct quotes in the text where they contribute to understanding. | See previous response. |
| 11. The results are already contextualised on the basis of the literature in this section. What is the new content that the discussion brings to this? A stronger separation of the presentation and discussion of results would be helpful. | We interpret this comment as a suggestion regarding reporting style. We consciously made the decision to take a narrative approach when reporting the findings in the result subsections in the results, and we acknowledge the overlap in sections at the onset. We also intentionally draw attention to the fact that various facets of the findings can be found in each vignette. This writing style aligns with the method of analysis we outlined in our article Roest, Milota & Leget (2021). |
| 12. Discussion Page 16: See Figure 1 and 2 for a visual representation of this change in perspective. - I don't understand the figures and what (additional) information they should provide. | We removed figure 1 and 2 on page 16 because they seem unhelpful to the readers. |
| 13. Subheadings could help the reader to follow your arguments. | We decided to keep the manuscript as it is, since we think that clarifications will make it easier to follow the argument. |
| 14. Clarify the scope (cancer patients) and discuss special features compared to other groups. | Please see our response to question one. |
